# Myeloid cell expression of the VDR and parathyroid hormone control the host response to influenza infection in mice

**DOI:** 10.3389/fimmu.2026.1939052

**Published:** 2026-09-30

**Authors:** Mengzhu Tang, Nicole E. Froelich, Nicholas J. Hanna, Veronika C. Weaver, Gustavo A. Nader, Troy C. Sutton, Margherita T. Cantorna

**Affiliations:** 1 Department of Veterinary and Biomedical Sciences, The Pennsylvania State University, University Park, PA, United States; 2 Department of Kinesiology, The Pennsylvania State University, University Park, PA, United States; 3 Huck Institutes of the Life Sciences, The Pennsylvania State University, University Park, PA, United States

**Keywords:** influenza virus, myeloid cells, parathyroid hormone, vitamin D, vitamin D receptor

## Abstract

**Introduction:**

Respiratory infections caused by seasonal influenza viruses remain a major global health burden. Vitamin D receptor (VDR) signaling regulates immune responses, but the role of the VDR and its interaction with parathyroid hormone (PTH) in host resistance to H1N1 influenza infection is not understood.

**Methods:**

Global VDR knockout (KO) mice, or myeloid (M) deletion of the VDR (M-VDR KO) and the respective wildtype (WT) littermates were infected with H1N1 influenza virus. Additional experiments treated WT mice with intermittent PTH or vehicle and then infected them with a H1N1 influenza A virus. Disease severity, mortality, viral burden and clearance, lung pathology, and inflammatory cytokine responses were evaluated.

**Results:**

H1N1 influenza infection of WT mice resulted in non-lethal disease, whereas VDR KO mice had 21% mortality, delayed viral clearance, more severe symptoms and lung pathology. The increased severity of respiratory disease was accompanied by higher type I and II interferon responses in the lungs. M-VDR KO mice had increased disease severity and delayed viral clearance compared to WT littermates. PTH-treated WT mice had increased clinical scores, influenza M gene, and cytokine responses compared to vehicle treated WT mice.

**Discussion:**

The work describes a novel paradigm in which the vitamin D-PTH axis governs host resistance to influenza virus disease by controlling the amount of inflammation and the amount of virus in the lung. In addition, the data suggest that VDR expression in myeloid cells is critical for controlling the early innate immune response to influenza viruses.

## Graphical abstract

**Figure:**
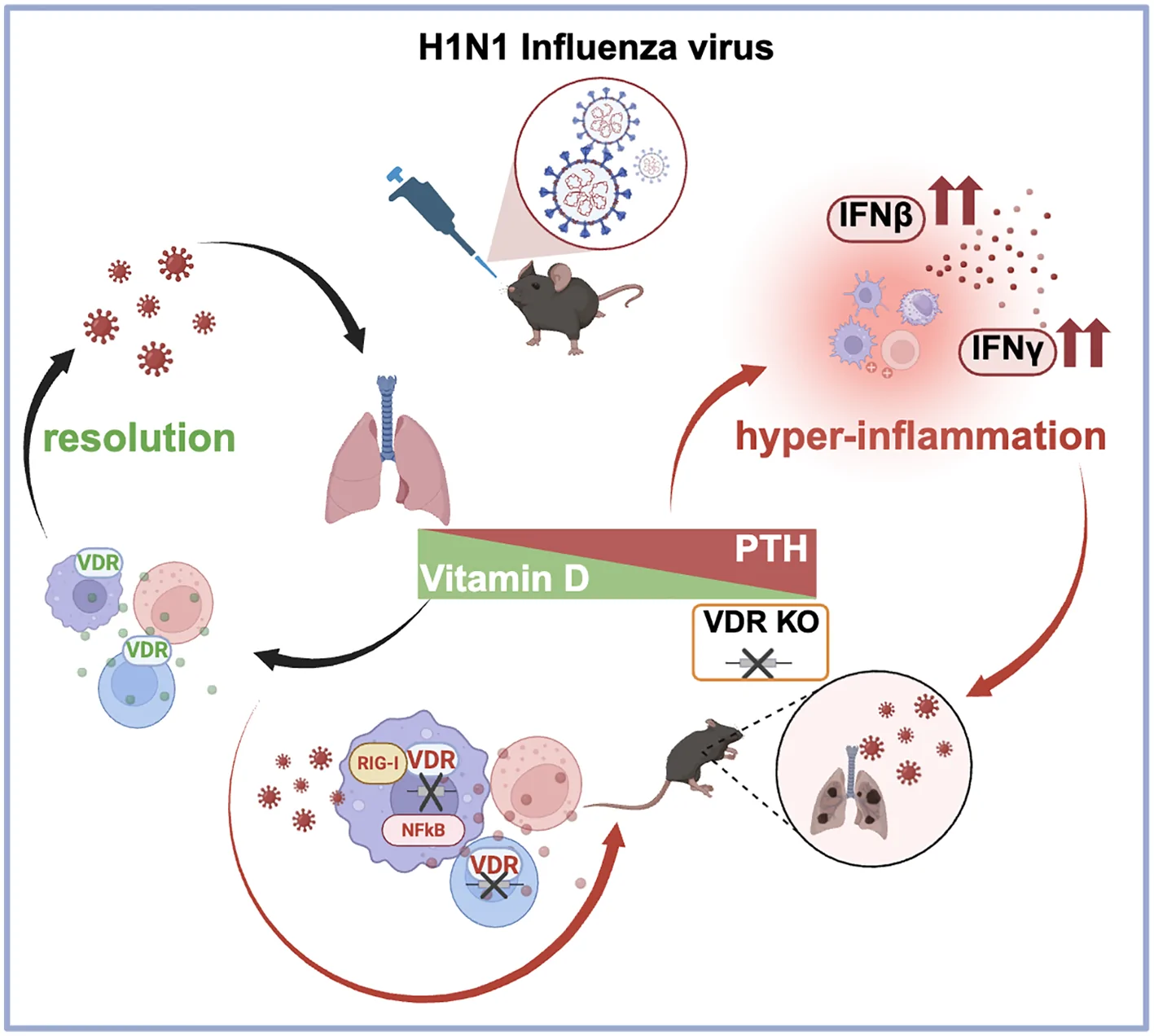

## Introduction

Seasonal and pandemic influenza viruses continue to pose significant global health challenges, contributing to millions of cases of severe respiratory illness and hundreds of thousands of deaths each year (1). Although vaccines and antiviral medications are available, their efficacy varies by strain and season. Disease severity during influenza virus infection is determined not only by viral pathogenicity, but also by the magnitude and regulation of host immune responses. While early innate antiviral signaling and interferon production are essential for viral clearance, excessive or prolonged inflammation can result in lung injury, impaired viral resolution, and severe disease (2). Identifying host pathways that balance protective versus pathogenic antiviral immunity is therefore critical for understanding susceptibility to severe influenza virus infection.

Vitamin D deficiency is highly prevalent worldwide and has been associated with increased susceptibility to respiratory infections, including influenza virus infection (3). Observational studies have demonstrated inverse correlations between circulating serum 25-hydroxyvitamin D [25(OH)D] levels and incidence of upper respiratory tract infections in both children and adults (4, 5). Furthermore, a large meta-analysis of randomized controlled trials demonstrated that vitamin D supplementation significantly reduced the risk of acute respiratory infections, particularly in individuals with profound vitamin D deficiency (6). However, clinical studies examining vitamin D supplementation during viral respiratory infections have produced inconsistent results, suggesting that the mechanisms by which vitamin D regulates antiviral immunity remain incompletely understood (7).

The biological effects of vitamin D are mediated through the vitamin D receptor (VDR), a nuclear receptor expressed in epithelial cells and multiple immune cell populations, including macrophages, dendritic cells, and activated T lymphocytes (8). The effect of VDR KO on host resistance to infection has been studied for bacterial and parasitic infections, but not viruses (9–12). Immune cells from VDR KO mice have been shown to overproduce inflammatory cytokines including TNF-α, IL-6, IFN-β1, IL-1β, IL-12, and IFN-γ that contribute to susceptibility to colitis, infection and autoimmunity (9–12). T cells from VDR KO mice proliferate more quickly and over- produce IFN-γ and IL-17 (9, 10). Because of the critical role of IFN-γ on host resistance to influenza virus by promoting antiviral immunity and viral clearance (11, 12), the effect of high IFN-γ on the host response of VDR KO mice to influenza infection is difficult to predict.

There is evidence that vitamin D may be a direct regulator of some viruses. Human bronchial epithelial cells have been shown to produce the vitamin D hydroxylase that produces the high affinity VDR binding compound, 1,25(OH)_2_D (13). 1,25(OH)_2_D treatment of human bronchial epithelial cells induced cathelicidin that suppressed respiratory syncytial virus replication *in vitro* (13). Although respiratory syncytial virus is a common respiratory illness that belongs to a different viral family than influenza virus, there is the possibility of direct effects of vitamin D on host responses that control viral replication.

An additional and largely unexplored component of vitamin D biology during infection is its close endocrine relationship with parathyroid hormone (PTH). PTH, a calcium-regulating peptide hormone, is secreted in response to low vitamin D status. PTH induces the production of the vitamin D hydroxylase to increase the circulating levels of 1,25(OH)_2_D (14). In a feedback loop, 1,25(OH)_2_D production inhibits PTH and together regulates serum calcium and phosphorous levels (14). Vitamin D deficiency and VDR KO result in very high PTH levels (15) even when calcium is normalized by rescue diets (16). PTH has recently been shown to be an immune system regulator (17–19). Importantly, PTH signaling enhanced T cell proliferation, and inflammatory cytokine production (e.g., TNF-α, IL-17) (17–19). Elevated PTH levels may contribute to the enhanced inflammation and cytokine production present in VDR KO mice.

VDR KO mice developed more severe and lethal influenza disease, characterized by heightened type I and II interferon production, with delayed viral clearance. Intermittent PTH treatment of WT mice exacerbated respiratory signs of influenza virus infection and resulted in higher viral M gene expression compared to vehicle-treated WT mice. PTH treated WT mice had greater IFN-γ production, elevated viral M gene and more severe clinical signs. Influenza infection of myeloid (M) specific, M-VDR KO mice, had impaired anti-viral responses and increased severity of symptoms. The increased susceptibility of the M-VDR KO mice demonstrates a critical role for myeloid cells as vitamin D targets in the host response to influenza. The combined effects of VDR KO and high PTH (16) result in excessive cytokine responses that are ineffective for host resistance to influenza viruses. Instead, the increased inflammation adds to the tissue injury. Interventions with vitamin D, would increase vitamin D status and reduce PTH, that potentially could reduce the impact of influenza virus infection.

## Materials and methods

### Mice

C57BL/6 VDR KO (VDRCre^+/+^, KO) and (VDRCre^-/-^) WT littermates were used for experiments as previously described (20). For myeloid (M)-specific deletion studies, VDR floxed mice (VDR^fl/fl^) were crossed with LysM-Cre transgenic mice (B6.129P2-Lyz2^tm1(cre)Ifo^/J), Jackson Laboratory, stock #004781) to generate M-VDR KO (VDR^fl/fl^ LysMCre^+^) and WT littermate controls. Genotyping primer sequences and expected PCR product sizes are provided in **Supplementary Table S1**. Experiments used 8–10 wk old mice of both sexes. There were no significant effects of sex on survival, body weight loss, and lethargy in WT and VDR KO mice and on respiratory symptoms in VDR KO mice following influenza infection (**Supplementary Figure 1**). There were significantly less respiratory symptoms in WT males late following the influenza infection than WT females (**Supplementary Figure 1**). Subsequent experiments were not powered to examine sex as a variable which is a limitation of the study. VDR KO and their WT littermates were fed vitamin D containing and high-lactose rescue diets (20% high lactose; Envigo, TD 96348, Madison, WI). All other experiments that used WT mice treated with PTH/vehicle or M-VDR KO and WT littermates used the same vitamin D containing diets but without the high-lactose (Envigo, TD 89124). All animal procedures were approved by the Institutional Animal Care and Use Committee (IACUC) at the Pennsylvania State University (University Park, PA).

### Serum calcium measurement

Serum calcium levels were measured using QuantiChrom^®^ colorimetric calcium assay kit (BioAssay Systems, DICA-500, Hayward, CA) according to the manufacturer’s instructions. Absorbance was measured at 612 nm using a microplate reader (Bio-Rad). Calcium concentrations were determined based on a standard curve generated with known calcium standards. Rescue diets were effective for normalizing serum calcium in VDR KO mice and there was no effect of PTH treatments on serum calcium levels in WT mice (**Supplementary Figure 2A**).

### Influenza virus

The recombinant mouse-adapted A/California/04/2009(H1N1pdm09) virus was rescued in a co-culture of human embryonic kidney 293T cells and Madin-Darby Canine Kidney (MDCK) cells using the 8-plasmid bi-directional reverse genetics system (21). HEK 293T cells (CRL-3216) and Madin-Darby Canine Kidney (MDCK) Cells, London Line, (FR-58) were obtained from the American Type Culture Collection, and International Reagent Resource, Influenza Division, WHO Collaborating Center for Surveillance, Epidemiology and Control of Influenza, Centers for Disease Control and Prevention, Atlanta, GA, USA, respectively. Both cell lines were maintained in in DMEM (Cytiva, SH30243.01, Marlborough, MA) supplemented with 10% fetal bovine serum (Seradigm, 1500-500, Radnor, PA), 25mM HEPES buffer (MDCK cells only), 2mM L-glutamine (Corning, 25-005-CI, Corning, NY) and 1% antibiotic-antimycotic (Gibco, 15240-062, Waltham, MA). All cells were cultured in a humidified incubator at 37°C with 5% CO_2_. After virus rescue, the virus was passaged once in MDCK cells to generate a virus stock. To generate a virus stock, MDCK cells were infected with virus diluted in Opti-MEM media (ThermoFisher, 31985-070, Waltham, MA) supplemented with 1 ug/mL of tosylsulfonyl phenylalanyl chloromethyl ketone (TPCK)-trypsin (Worthington Biochemical, LS003740, Lakewood, NJ). Forty-eight to 72 hours post-infection, when cytopathic effect was visible, the cell supernatant was collected, centrifuged at 750 x g for 10 minutes, aliquoted, and frozen at -80°C until use.

### Influenza virus infection

Mice were anesthetized with isoflurane and intranasally infected with 30 TCID_50_ of mouse-adapted influenza A/H1N1 (A/California/04/2009(H1N1pdm09) virus in 50μL of sterile Gibco’s reduced-serum minimum essential media (ThermoFisher, 31985-070, Waltham, MA), as described (21). Mice were monitored daily for survival, body weight loss, and clinical signs, including respiratory distress and lethargy, for up to 14 days post-infection. Mice reaching predetermined humane endpoints (extreme respiratory distress and/or reaching 25% body weight loss) were sacrificed and recorded as having succumbed to the infection. Clinical symptoms of H1N1 influenza virus infection were scored blindly and included weight loss, respiratory scores, and lethargy scores [0 no symptoms to 3 labored breathing/lack of movement (21)]. Respiratory scoring was based on nasal flaring, labored breathing, and wheezing. Lethargy scoring considered activity level.

### Histopathology

Lung tissues were fixed in 10% formalin, paraffin-embedded, sectioned, and stained with hematoxylin and eosin (H&E) by the PSU Animal Diagnostic Lab Histology lab. Lung pathology was scored blindly (scale 0–3: total of 9) for lymphocyte infiltration, perivascular and bronchial cuffing, and alveolar hemorrhage (21). The histopathologic evaluation of tissue sections was performed using criteria previously established for H1N1-infected mouse lungs.

### RNA isolation and quantitative PCR

Tissues were homogenized in TRIzol reagent (Sigma-Aldrich, T9424, St. Louis, MO) and total RNA was extracted using chloroform-isopropanol precipitation. RNA concentration and purity were assessed using a NanoDrop spectrophotometer (ThermoFisher Scientific, Waltham, MA). 2 µg of total RNA per sample was reverse transcribed into cDNA using Reverse Transcriptase (Promega, Madison, WI). Gene-specific primers were obtained from Integrated DNA Technologies (IDT, Coralville, IA) and are listed in **Supplementary Table S1**. Quantitative PCR was performed using SYBR Green Master Mix (Azura Genomics, Raynham, MA) on a StepOne Plus Real-Time PCR System (Applied Biosystems, Carlsbad, CA). Relative gene expression levels were calculated using the 2^^−ΔΔCT^ method, with GAPDH as the housekeeping gene. GAPDH Ct values were tightly clustered between 16 and 18 indicating that the cDNA was robust and highly abundant minimizing technical noise. GAPDH stability was verified by standard deviations of <0.5 across genotypes, target genes and time points in our experiments and was used as the only reference gene for qPCR normalization. All values were normalized to uninfected or vehicle treated WT samples.

### Virus quantitation

Influenza virus M gene was quantitated by qRT-PCR as described above using M gene specific primers (**Supplementary Table S1**). Infectious virus was quantified using the Tissue Culture Infectious Dose 50% (TCID_50_) assay, a standard method for determining the concentration of infectious virus based on cytopathic effect in cell culture. 100mg of lung tissue was weighed and flash-frozen in liquid nitrogen, then stored at −80 °C until processing. Lung tissues were homogenized in 1 mL of Opti-MEM supplemented with antibiotic-antimycotic solution (Corning, 30-004-CI, Glendale, AZ; final concentrations: 100 U/mL penicillin, 100 µg/mL streptomycin, and 0.25 µg/mL amphotericin B). Homogenates were centrifuged at 750 xg for 10 minutes at 4 °C. Serial 10-fold dilutions (8 replicates per dilution) were added (20μl) to MDCK cells seeded into a 96-well plate. Plates were evaluated for cytopathic effects using an inverted microscope at 10x magnification. Cytopathic changes were scored based on characteristic morphological alterations in MDCK cells, including cell rounding, detachment, monolayer disruption, and cell clumping. Calculations were done as described previously (21, 22).

### Gastrocnemius muscle

Left and right gastrocnemius muscles were isolated, rinsed in cold PBS, blotted on lint-free paper, and weighed. Left and right muscle mass was averaged for each animal and normalized to terminal body weight (mg/g). Percent muscle loss was calculated relative to the mean value from uninfected (d0) genotype-matched controls using: % loss = [1 − (mg/g infected ÷ mean mg/g uninfected)] × 100.

### *In vivo* PTH treatment

WT mice received daily subcutaneous injections of 50µl of human PTH residues 1–34 which is highly conserved in mammals and cross-reactive (PTH 1-34, Bachem, 52232-67-4, Torrance, CA) in PBS at a dose of 80 μg/kg or PBS (vehicle) beginning on the day of influenza virus infection as described (23). Mice were randomly allocated to the vehicle or PTH treatment groups. All mice were housed under identical conditions.

### Cell isolation

Spleens were harvested and processed into single-cell suspensions by mechanical disruption through a 70 µm cell strainer. Red blood cells were lysed using lysis buffer, and viable splenocytes were resuspended in complete RPMI 1640 medium. Lungs were collected from euthanized mice in calcium- and magnesium-free HBSS and minced into small fragments. Tissues were first incubated in HBSS with 0.5M EDTA and 1M dithiothreitol at 37 °C for 30 minutes with stirring to remove mucus. After centrifugation at 600 xg for 10 minutes at 4 °C, tissue fragments were digested in RPMI-C media containing 230 U/mg collagenase type I (Worthington, LS004197, Lakewood, NJ) and DNase I (Sigma-Aldrich, D4513, St. Louis, MO) at 37 °C for 1h in a shaking incubator. The resulting cell suspension was filtered through a 70 μm cell strainer and washed with PBS. Red blood cells were lysed using RBC lysis buffer, followed by centrifugation and resuspension in RPMI-C medium. Cell viability and counts were determined using a hemocytometer and trypan blue.

### *In vitro* culture and treatments

Splenocytes were seeded in 24-well plates at a density of 1× 10^6^ cells/mL and stimulated with plate-bound anti-CD3 antibody (10 µg/mL, Tonbo Biosciences, clone 145-2C11, San Diego, CA). Experimental groups received either: 1) No treatment, 2) Recombinant human parathyroid hormone (PTH 1-34; 5 nM), or 3) 1,25-dihydroxyvitamin D_3_ (1,25(OH)_2_D; 25 nM). PTH was freshly diluted and added every 12 hours during the 48h culture period.

### Flow cytometry

A list of the Abs is described in in **Supplementary Table S1**. For intracellular staining, cells were resuspended at 2x10^6^ cells/mL in RPMI-C (pH 7.2) and stimulated with 2 μg/mL Brefeldin A (BioLegend, 420601, San Diego, CA), followed by fixation and then permeabilized using the FoxP3/Transcription Factor Staining Buffer Set (eBioscience, 00-5523-00, Carlsbad, CA) and stained with intracellular Abs in permeabilization buffer overnight at 4 °C. All samples were acquired on a 4L (16V-14B-10YG-8R) Cytek^®^ Aurora (Pennsylvania State University Flow Cytometry Core Facility) and analyzed with FlowJo software version 10.10 (BD Biosciences, Ashland, OR). Single-positive compensation beads (Biolegend, 424601/424602, San Diego, CA) and unstained controls were included to establish gating strategies. The flow cytometric markers used to identify each immune cell population are provided in **Supplementary Table S1**. Representative gating strategy for identifying lymphocytes, CD4^+^, CD8^+^ T cells and intracellular cytokine staining can be found in (**Supplementary Figure 3**).

### Enzyme-linked immunosorbent assay

Influenza-specific serum IgG was quantified by indirect ELISA using influenza cell lysate–coated 96-well plates. Briefly, plates were coated with influenza cell lysates (1.2 mg/mL; 5 µg/well in 50 µL per well) and incubated overnight at 4 °C. Plates were washed, then blocked with 150 µL per well of 5% non-fat milk for 1 h at 37 °C, followed by three washes. Mouse sera were prepared as 2-fold serial dilutions starting at 1:10 in 1× PBS, and 50 µL per well of each dilution was added to the plate. Bound IgG was detected using an HRP-conjugated goat anti-mouse IgG (H+L) secondary antibody (Thermo Fisher Scientific, 31430, Waltham, MA) diluted 1:2,000 in 1× PBS, added at 50 µL per well, and incubated for 1 h at 37 °C. Plates were washed three times with PBST and developed with 100 µL per well of substrate for 25 min at room temperature. The reaction was stopped by adding 50 µL per well of 1 M sulfuric acid directly to each well, and absorbance was read at 450 nm using a Bio-Rad microplate reader.

### Bone marrow-derived macrophage isolation

Bone marrow cells were isolated from femurs and tibias of WT, VDR KO, and M-VDR KO mice and differentiated into BMDM by culture in complete RPMI 1640 medium (Gibco, 11875-093, Waltham, MA) supplemented with 10% fetal bovine serum (FBS), 2 mM L-glutamine, and 1% antibiotic–antimycotic. L929 cell–conditioned medium (20–30%) was added for 6d to promote macrophage differentiation. L929 fibroblasts (ATCC, CCL-1, Manassas, VA) were cultured in DMEM supplemented with 10% FBS until confluence. Supernatants were collected at d7, centrifuged to remove debris, filtered through a 0.22 μm filter (Millipore Sigma, SLGP033RS, Burlington, MA), aliquoted, and stored at −20 °C until use.

### VDR protein detection by western blot

Total protein lysates were prepared from kidney and BMDM of WT, M-VDR KO and VDR KO mice using RIPA buffer (Thermo Fisher Scientific, 89901, Waltham, MA) supplemented with protease inhibitor cocktail (Sigma-Aldrich, P8340, St. Louis, MO). Protein concentrations were determined using a bicinchoninic acid (BCA) protein assay kit (Thermo Fisher Scientific, 23225, Waltham, MA). Equal amounts of protein (20 μg) were separated on 10% SDS-PAGE gels and transferred onto PVDF membranes (MilliporeSigma, IPVH00010, Burlington, MA). Membranes were blocked in 5% skim milk in TBST for 2 h and incubated overnight at 4 °C with primary antibodies against VDR (mouse monoclonal, Santa Cruz Biotechnology, sc-13133, Dallas, TX; 1:100 dilution) (24) and GAPDH (rat monoclonal, BioLegend, W17079A, San Diego, CA; 1:10,000 dilution). Protein bands were visualized using enhanced chemiluminescence substrate (Thermo Fisher Scientific, SuperSignal™ West Femto, 34094, Waltham, MA) and imaged using an Azure 600 imaging system (Azure Biosystems, Dublin, CA). The BMDM have a nonspecific higher MW band present in all samples. The VDR band is 53kDa and is indicated by the red arrow (**Supplementary Figure 6A**). WT mice have VDR protein expression in kidney and BMDM, while the M-VDR KO mice have VDR protein in the kidney but not the BMDM and VDR KO mice have no VDR protein in either the kidney or BMDM (**Supplementary Figure 6A**).

### Statistical analysis

Values are expressed as mean ± SEM. Statistical analyses were performed using GraphPad Prism version 10 (GraphPad Software, San Diego, CA). Data were mice from two to three independent experiments (with the exception of viral titers which was from one experiment), with n = 3–8 mice per group and timepoints. Sample sizes were based on prior influenza infection experiments and available animal cohorts.

For comparisons involving two independent groups, statistical significance was assessed using an unpaired two-tailed Student’s t test. Experiments involving two independent variables (genotype and time) were analyzed using two-way ANOVA, and the main effects of genotype and time and the genotype × time interaction are reported where applicable. When all pairwise group comparisons were performed, Tukey’s multiple-comparisons test was used for *post hoc* analysis. For comparisons between genotypes at individual time points, multiple comparisons were controlled using the two-stage step-up Benjamini, Krieger, and Yekutieli false discovery rate (FDR) procedure (Q = 0.05).

Outliers were identified using the ROUT method in GraphPad Prism and excluded prior to statistical analysis where applicable. Data normality was assessed using the Shapiro–Wilk test. When data did not meet the assumptions of normality, the Mann–Whitney U test was used. Survival curves were analyzed using the log-rank (Mantel–Cox) test. A p < 0.05 was considered statistically significant.

## Results

### VDR KO mice develop lethal H1N1 influenza virus infection

To determine the role of the VDR in host resistance to influenza virus infection, WT and VDR KO mice were infected with 30 TCID_50_ H1N1 influenza virus. At this dose of H1N1 influenza virus, none of the 36 WT mice reached endpoint criteria (**Figure 1A**). Conversely, VDR KO mice had 79% survival rates (n=22 of 28 survived to d14), which were significantly less than the survival of WT mice (**Figure 1A**). Both WT and VDR KO mice lost about 10% of their starting body weight and body weight loss peaked at d8 post-infection with the surviving mice recovering their body weight by d12 post-infection (**Figure 1B**). VDR KO mice infected with 30 TCID_50_ H1N1 virus had 8.3% decrease in gastrocnemius muscle mass at d9 post-infection (**Supplementary Figure 2B**), however, the muscle wasting was not seen in WT mice infected with 30 TCID_50_ H1N1 virus. There was no difference between WT and VDR KO gastrocnemius muscle mass at baseline. Respiratory distress and lethargy scores started earlier post-infection (d3) and were significantly more severe in VDR KO than WT mice (**Figures 1C, D**, *p* < 0.0001). Influenza viral M gene expression was detected in the lung from d3-d9 post-infection and M gene expression was significantly higher in VDR KO lung than WT lung (**Figure 2A**; d3 and d9, *p* < 0.05). TCID_50_ assays for infectious H1N1 influenza virus confirmed the higher titers of virus in the VDR KO mouse lungs compared to WT mouse lungs at d6 post-infection (**Figure 2B**; *p* < 0.01). Histopathology showed that the WT mouse lungs had less perivascular and alveolar inflammation than VDR KO mouse lungs at d9 post-infection, resulting in total pathology scores that were significantly higher for VDR KO than WT mouse lungs (**Figures 2C–E**, *p* < 0.05). Collectively, the data show that VDR KO mice are more susceptible to H1N1 influenza virus disease than their WT littermates.

**Figure 1:**
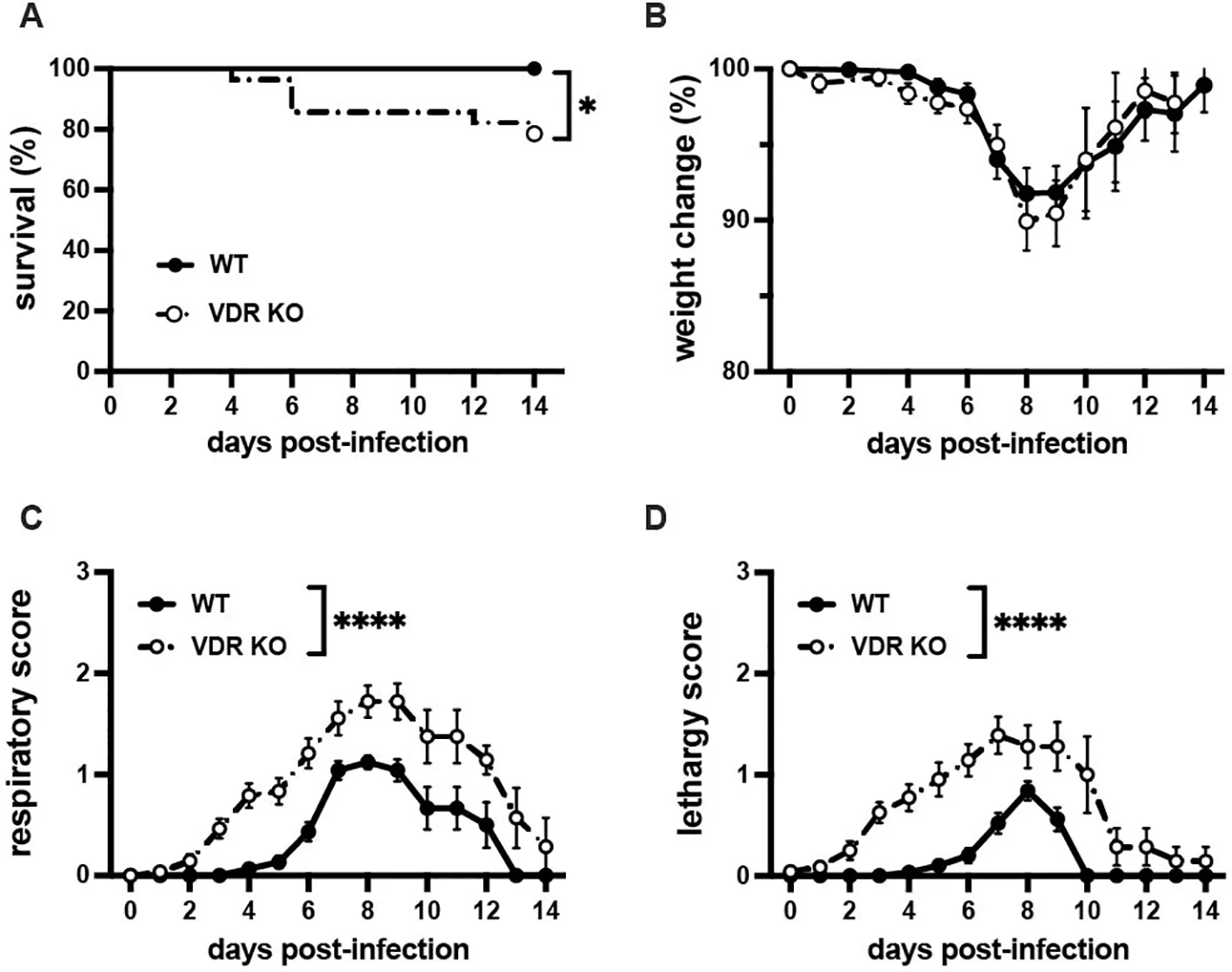
VDR KO exacerbates disease severity following influenza virus infection. WT and VDR KO mice were infected intranasally with H1N1 influenza virus and monitored over a 14-day period. **(A)** Kaplan–Meier survival curves, **(B)** body weight change, **(C)** respiratory scores, and **(D)** lethargy scores were recorded daily post-infection. Values are the mean ± SEM and represent pooled results from two to three independent experiments (WT: n = 36; VDR KO: n = 28 total). Statistical significance was determined using the log-rank (Mantel-Cox) test for survival **(A)** and two-way ANOVA with Tukey’s multiple comparisons test adjusted using the two-stage step-up Benjamini, Krieger, and Yekutieli false discovery rate (FDR) procedure for **(B–D)**. *p < 0.05; ****p < 0.0001.

**Figure 2:**
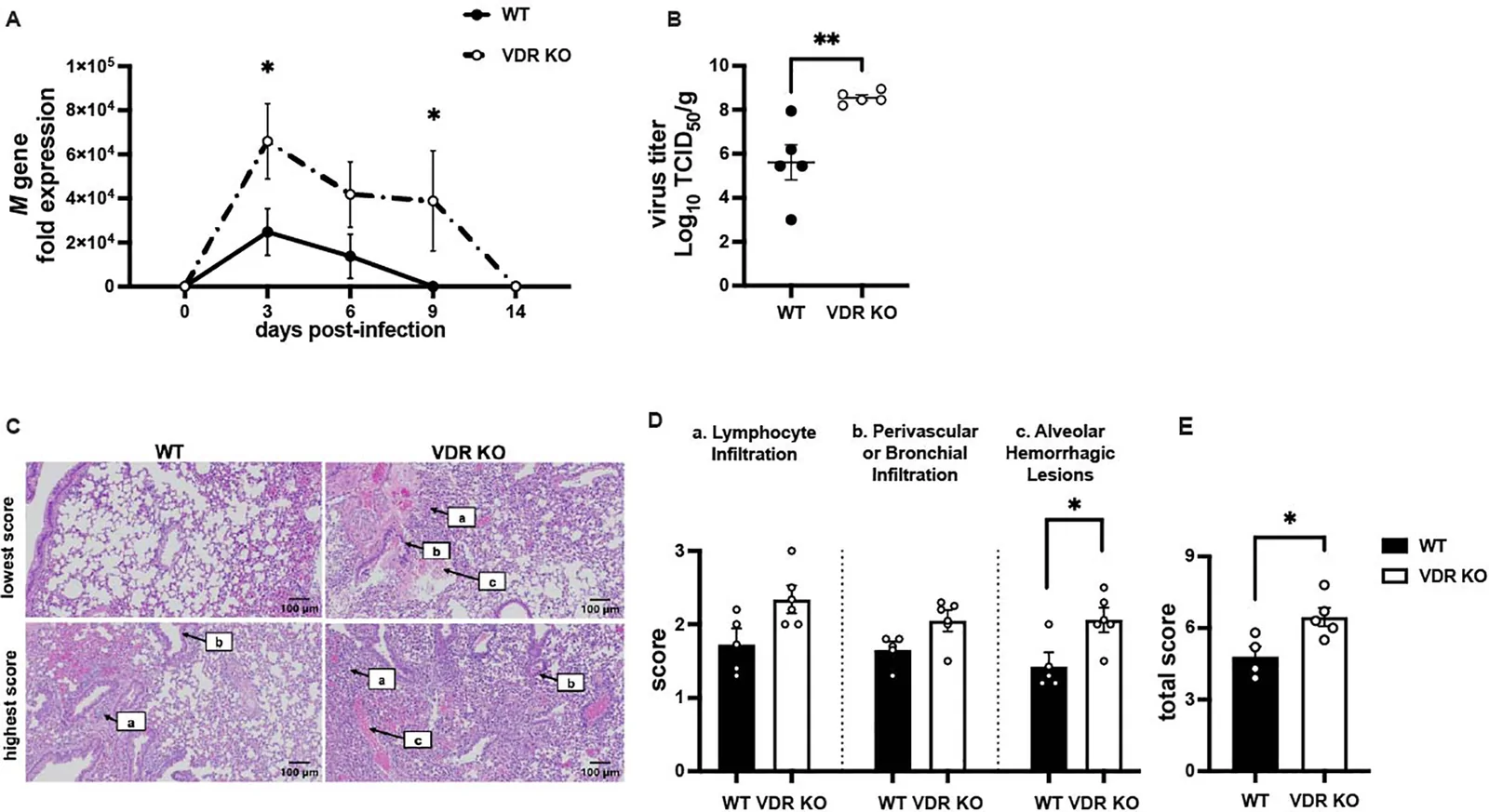
Increased viral burden and pathology in VDR KO mice. Lungs from influenza virus infected WT and VDR KO mice. **(A)** Influenza viral matrix (*M*) gene expression (n = 3 for (d0), n=7 mice/all other time points, 3 experiments), **(B)** viral titers (log_10_ TCID_50_/g lung tissue) on d6 post-infection (n=5 per group, 1 experiment), **(C)** Representative images are shown from lungs from WT and VDR KO mice at d9 post-infection, with the lowest (top row) and highest (bottom row) inflammation scores within each genotype (n = 5–6 mice/group, 2 experiments). Pathologic features are labeled as follows: (a) lymphocyte infiltration, (b) perivascular and peribronchiolar cuffing, and (c) alveolar hemorrhage. Images taken at 40X magnification; scale bars = 100 μm, **(D)** histopathological scoring (scale 0-3), and **(E)** quantification of total lung pathology scores (scale 0-9). Values are the mean ± SEM from one to three independent experiments. Each symbol represents an individual mouse. Statistical analysis was performed using two-way ANOVA with Tukey’s multiple comparisons test for A, and unpaired Student’s t-tests for **(B–D)** *p < 0.05; **p < 0.01.

### Overproduction of cytokines in the VDR KO lung following H1N1 infection

Uninfected (d0), lungs from WT and VDR KO mice expressed very low levels of *Ddx58* (viral RNA sensor, *RIG-I), Nfkb1, Ifn-β, Il-6, Il-17a, Ifn-γ*, and *Il-10* mRNA (**Figure 3**). In contrast, uninfected VDR KO lungs had high expression of *Tnf-α*, indicating an increased inflammatory tone prior to infection (**Figure 3D**, *p* < 0.001). *Ddx58* expression was induced following H1N1 infection in both WT and VDR KO lungs, with VDR KO mice having significantly higher expression at d6 and d9 post-infection compared with WT mice (**Figure 3A**, *p* < 0.05). Similarly, *Nfkb1* transcript levels were significantly higher in VDR KO lungs at d6 and d9 (**Figure 3B**, *p* < 0.01). Peak cytokine responses were at d3 for *Ifn-β*, *Il-6, Il-17a* and *Tnf-α or d6 for Ifn-γ* and *Il-10* (**Figure 3**)*. Ifn-β* mRNA levels were significantly higher (10-fold more) in VDR KO compared to WT lungs at d3 post-infection (**Figure 3C**, *p* < 0.0001). *Ifn-β* resolved to baseline values by d9 post-infection in WT and in the surviving VDR KO mice (**Figure 3C**). Infection induced *Tnf-α* expression in WT lungs, whereas Tnf-α transcript levels in VDR KO lungs remained elevated but were not further increased from the uninfected values (**Figure 3D**). *Il-*6 mRNA was still detectable in VDR KO mice at d9, while WT resolved to baseline by d9 (not significantly different, **Figure 3E**). Both VDR KO and WT resolved the *Il-6* response by d14 post-infection (**Figure 3E**). *Ifn-γ* mRNA peaked at d6 post-infection and resolved back to baseline in both WT and VDR KO mice (**Figure 3F**). *Il-17a* mRNA was relatively low in the lung throughout the infection of both WT and VDR KO mice (**Figure 3G**). *Il-10* mRNA expression was induced to the same level in the lungs of WT and VDR KO mice from d3-d9 post-H1N1 infection (**Figure 3H**). At d14 post-infection the VDR KO lung had significantly higher *Il-10* mRNA expression compared to WT lung (**Figure 3H**, *p* < 0.05). VDR KO mice had higher expression of *Ddx58, Nfkb1, Ifn-β*, and *Tnf-α* during the acute phase of the influenza infection, with increased *Il-10* expression later at d14 as compared with WT mice.

**Figure 3:**
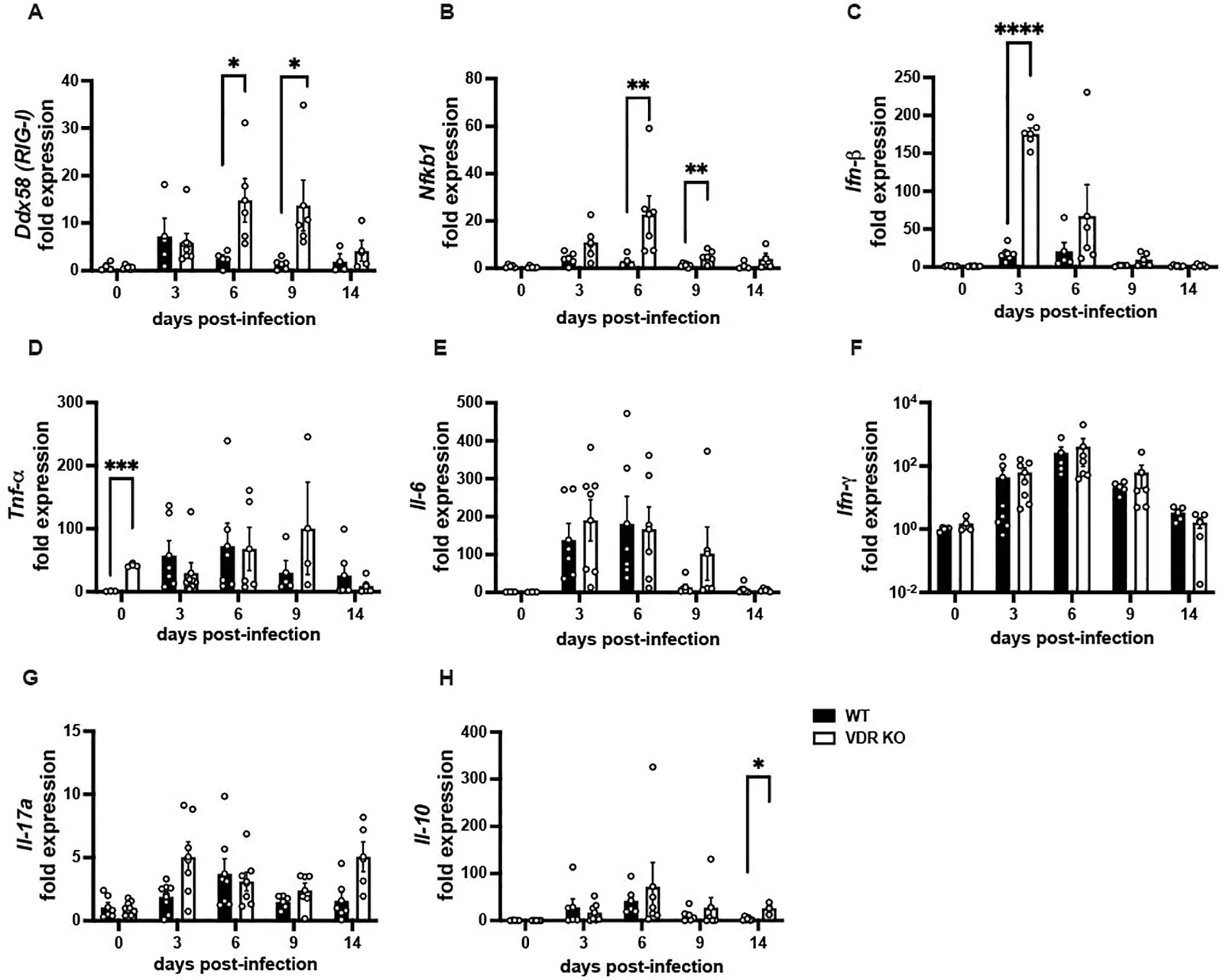
VDR KO alters innate antiviral signaling and inflammatory cytokine gene expression in the lungs during H1N1 infection. Relative mRNA expression of **(A)** *Ddx58* (RIG-I), **(B)** *Nfkb1*, **(C)** *Ifn-β*, **(D)** *Tnf-α*, **(E)** *Il-6*, **(F)** *Ifn-γ*, **(G)** *Il-17A*, and **(H)** *Il-10* was quantified by qPCR and normalized to housekeeping genes. Values are reported as fold expression relative to uninfected WT controls. Values are the mean ± SEM from d0 n=3-5 (1 experiment) and 3 independent infection experiments (n = 4–8 mice per time point in each group). Each symbol represents an individual mouse. Statistical analysis was performed using two-way ANOVA with Tukey’s multiple comparisons test and were adjusted using the two-stage step-up Benjamini, Krieger, and Yekutieli false FDR procedure. *p < 0.05; **p < 0.01; ****p < 0.0001.

### Increased cell infiltrates and IFN-γ production in lungs from influenza A virus infected VDR KO mice

Lungs from uninfected WT and VDR KO mice had low frequencies and absolute numbers of inflammatory myeloid, NK, B, CD4^+^, and CD8^+^ T cells (d0 values in **Figure 4**). Infection resulted in robust recruitment of all cell types to the lungs of both WT and VDR KO mice (**Figure 4**). VDR KO lungs had significantly higher frequencies and/or total numbers of myeloid, CD4^+^ and CD8^+^ T cells than WT lungs at d9 post-infection (**Figure 4**, *p* < 0.05). In contrast, NK cell frequencies but not total cell counts were lower in VDR KO lungs than WT at d9 post-infection (**Figures 4D, E**, *p* < 0.05). The difference between NK cell frequencies and cell counts is likely due to the expansion of over cell types. B cell frequencies, cell counts, and serum anti-influenza IgG responses were not different between genotypes (**Figures 4F, G**, **Supplementary Figure 2C**). VDR KO lungs had higher IFN-γ secreting cells than WT lungs at d9 post-infection (**Figures 5A, B**, *p* < 0.001). IFN-γ secretion from CD4^+^ and CD8^+^ T cells contributed to the higher total IFN-γ in the VDR KO lung (**Figures 5C–F**, *p* < 0.05). Overall, VDR KO mice had more myeloid and CD4^+^ T-cell infiltrates and higher IFN-γ production than WT mice following influenza virus infection.

**Figure 4:**
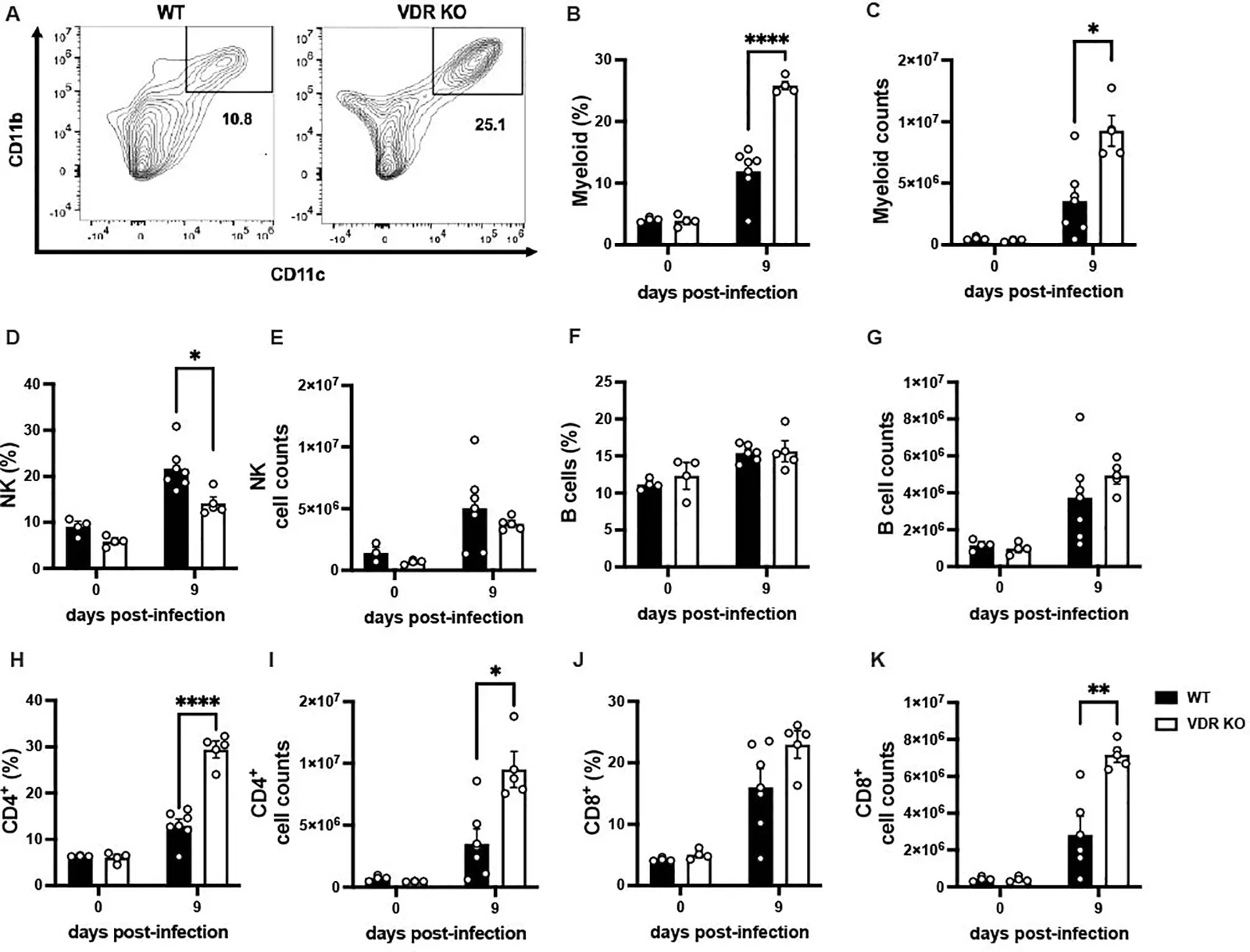
VDR KO affects the immune cell infiltrates in the lung. **(A)** Representative flow cytometry plots showing myeloid gating (MHCII^+^CD11b^+^CD11c^+^), **(B)** frequencies and **(C)** cell number of myeloid cells, **(D)** frequencies and **(E)** cell numbers of NK cells, **(F)** frequencies and **(G)** cell number of B cells, **(H)** frequencies and **(I)** cell number of CD4^+^ cells and **(J)** frequencies and **(K)** cell number of CD8^+^ cells. Values are the mean ± SEM from two independent experiments (n = 4–8 mice per time point per group). Each symbol represents an individual mouse. Statistical significance was determined using two-way ANOVA with Tukey’s multiple comparisons test. *p < 0.05; **p < 0.01; ****p < 0.0001.

**Figure 5:**
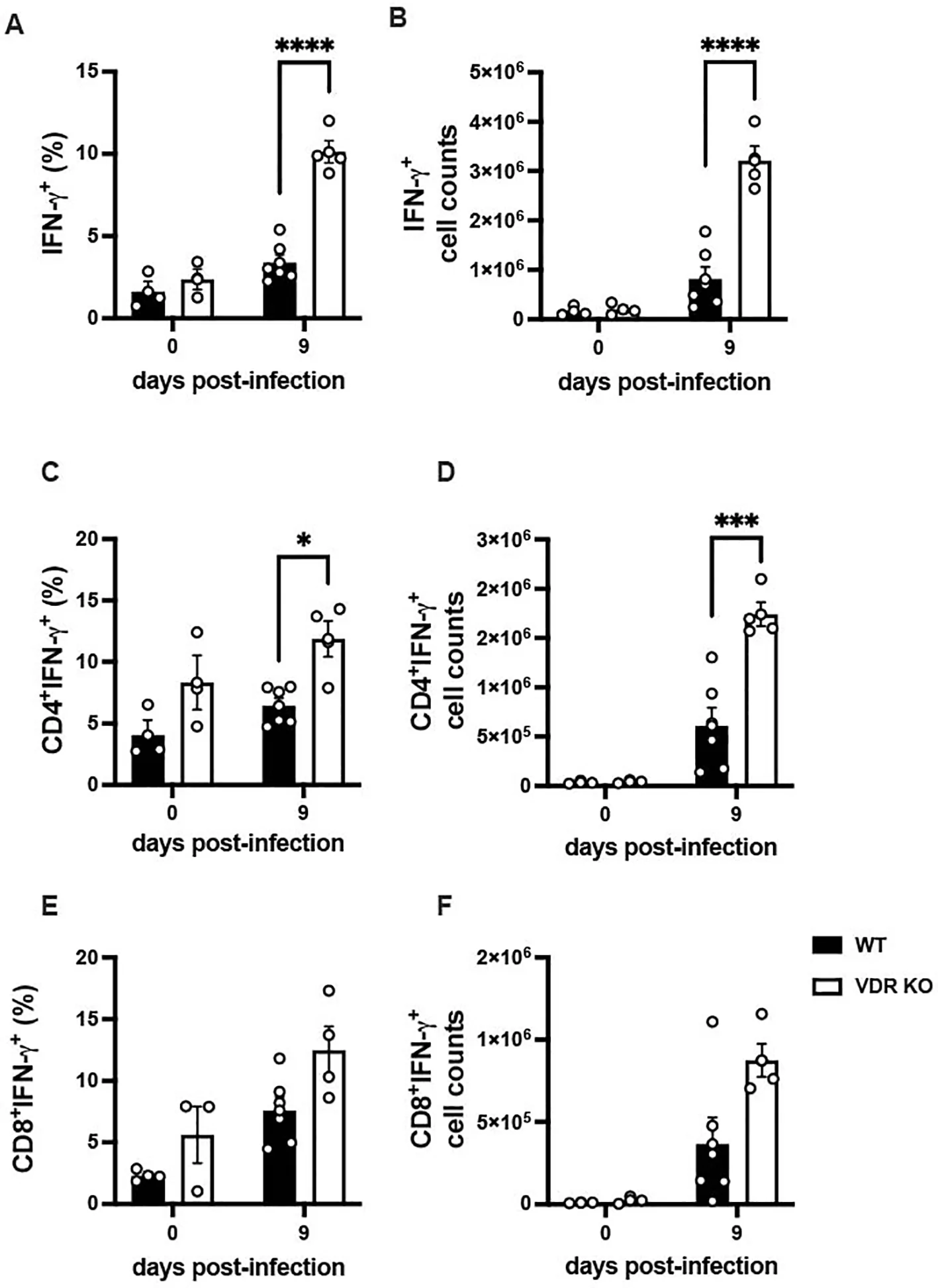
Increased IFN-γ responses in the lungs of VDR KO mice following H1N1 infection. Frequencies and absolute numbers of **(A, B)** total IFN-γ^+^ cells, **(C, D)** CD4^+^IFN-γ*^+^* T cells, **(E, F)** CD8^+^IFN-γ^+^ T cells. Values are the mean ± SEM from two independent experiments (n = 4–8 mice per time point per group). Each symbol represents an individual mouse. Statistical analysis was performed using two-way ANOVA with Tukey’s multiple comparison test. *p < 0.05; ***p < 0.001; ****p < 0.0001.

### PTH exacerbates WT host resistance to H1N1 infection

PTH treatment of WT mice increased renal *Cyp27b1* mRNA expression confirming the documented effects of PTH on the vitamin D 1-alpha hydroxylase ( (16, 25, 26), **Figure 6A**, *p* < 0.05). Intermittent PTH treated WT and vehicle-treated WT mice lost the same amounts of body weight without any mortality following H1N1 infection, with no lethality (**Supplementary Figures 4A, B**). PTH-treated WT mice had higher respiratory and lethargy scores than vehicle-treated WT mice following influenza virus infection (**Figures 6B, C**, *p* < 0.05). In addition, viral M gene expression in the lungs was significantly higher in PTH-treated mice at d6 post-infection compared to vehicle-treated mice (**Figure 6D**, *p* < 0.0001). Infection induced the recruitment of myeloid, B, NK and T cells into the lung of both vehicle and PTH-treated WT mice (**Supplementary Figures 4C–G**). PTH-treated mice had higher frequencies (**Figure 6E**, *p* < 0.01) and total numbers (**Supplementary Figure 5A**, *p* < 0.01) of IFN-γ^+^ cells in the lung at d6 and d9 post-infection than vehicle treated controls. Both vehicle- and PTH-treated WT mice had similar robust increases in CD4^+^IFN-γ^+^ and CD8^+^IFN-γ^+^ cells (**Supplementary Figures 5B, C**). Influenza virus infection induced more Th17 cells or double positive Th1/Th17 cells than Th1 following influenza virus infection of either WT or PTH treated WT mice (**Supplementary Figures 5D–G**). PTH treatment significantly increased the frequency of FoxP3+ Tregs at d9 compared to d6, however the frequency of Tregs were not different at d9 between vehicle- and PTH- treated WT mice (**Supplementary Figure 5H**). *In vitro*, 1,25(OH)_2_D or PTH treatments of anti-CD3 stimulated splenocytes resulted in higher frequences of CD4^+^CD25^+^ T cells compared to the untreated controls (**Supplementary Figure 5I**, *p* < 0.0001). The effect of PTH was significantly higher than the effect of 1,25(OH)_2_D on CD4^+^CD25^+^ expression (**Supplementary Figure 5I**, *p* < 0.0001). Intermittent PTH treatments were associated with higher IFN-γ+ and the increased severity of influenza virus infection.

**Figure 6:**
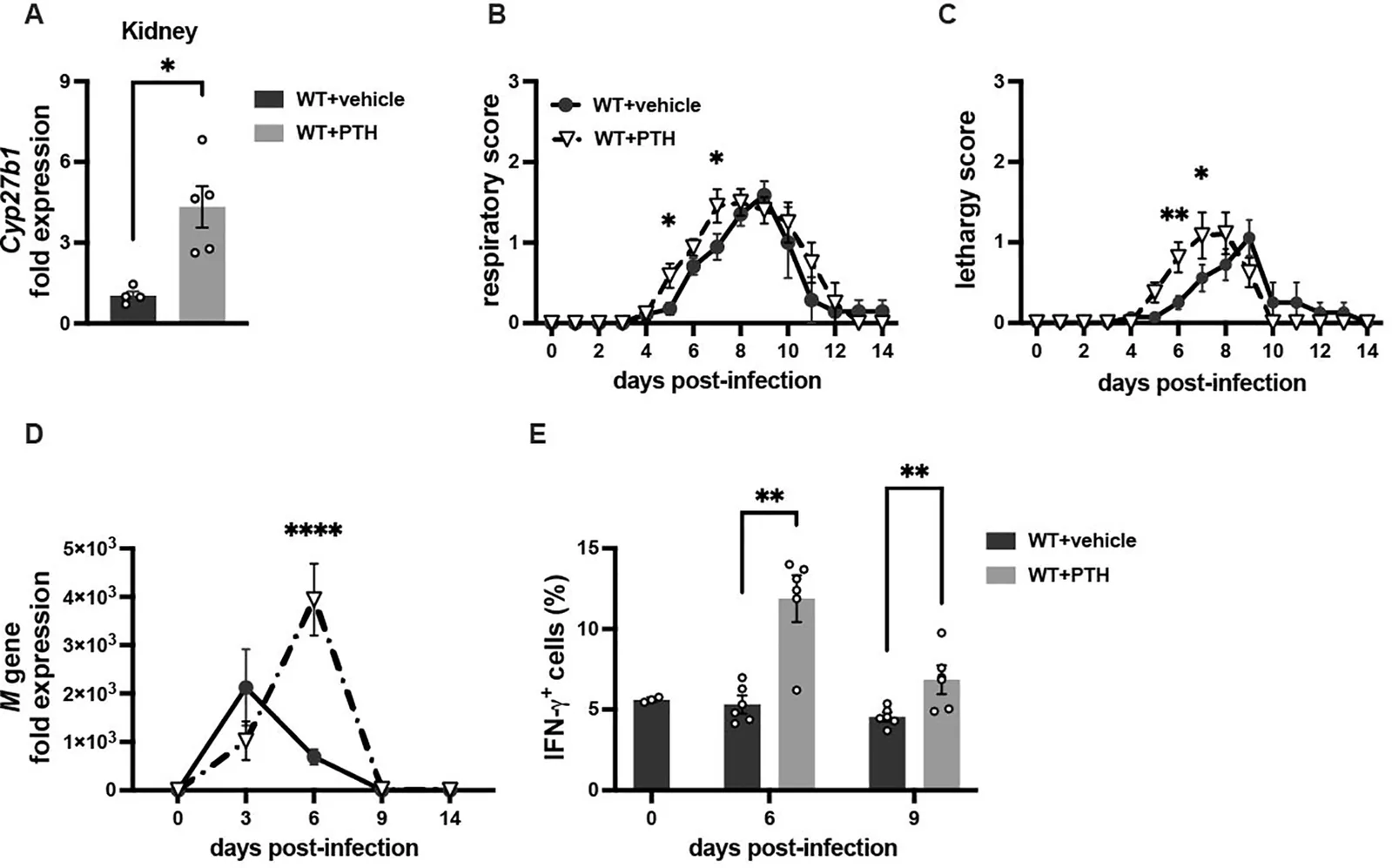
PTH treatments increased disease severity following influenza virus infection. **(A)** Relative Cyp27b1 mRNA expression in the kidney of uninfected mice 3h following PTH treatment (n = 4 mice/group, 1 experiment), **(B)** respiratory scores (vehicle WT n = 30, PTH-treated WT n = 22, 3 experiments), **(C)** lethargy scores (vehicle WT n = 30, PTH-treated WT n = 22, 3 experiments), **(D)** influenza viral *M* gene expression (n = 3 for (d0), n=7 mice/all other time points, 3 experiments), and **(E)** frequency of IFN-γ^+^ T cells in the lungs (n = 3–6 mice per time point per group, 2 experiments). Values are the mean ± SEM, pooled from one to three independent experiments. Each symbol represents an individual mouse. Statistical significance was assessed using two-way ANOVA with Tukey’s multiple comparisons test. *p < 0.05; **p < 0.01; ****p < 0.0001.

### Increased susceptibility of M-VDR KO mice to influenza infection

M-VDR KO mice have myeloid cell specific KO of the VDR (**Supplementary Figure 6**). Because the VDR is only knocked out in myeloid cells and not the kidney (**Supplementary Figure 6**), PTH levels are the same in M-VDR KO and WT littermate mice (27). The M-VDR KO and their WT littermates all survived the influenza infection (**Supplementary Figure 6B**). M-VDR KO mice had significantly more weight loss, with higher respiratory and lethargy scores compared to WT littermates (**Figures 7A–C**, *p* < 0.05). In addition, the M-VDR KO lungs had more viral M gene expressed at d6 post-infection compared to WT mice at d6, consistent with impaired viral clearance (**Figure 7D**, *p* < 0.05). The M-VDR KO mice had significantly higher expression of Ddx58 at d9 (**Figures 7E**, RIG-I, innate antiviral signaling gene), and showed prolonged *Nfkβ1*expression that was not significantly different than WT (**Figure 7F**). *Tnf-α, IL-6*, *Ifn-β* and Ifn*-γ*, mRNA expression was the same in the lung of M-VDR KO and WT mice over the course of infection (**Supplementary Figures 6C–F**). VDR expression in myeloid cells is required for efficient viral control and regulation of innate immune signaling.

**Figure 7:**
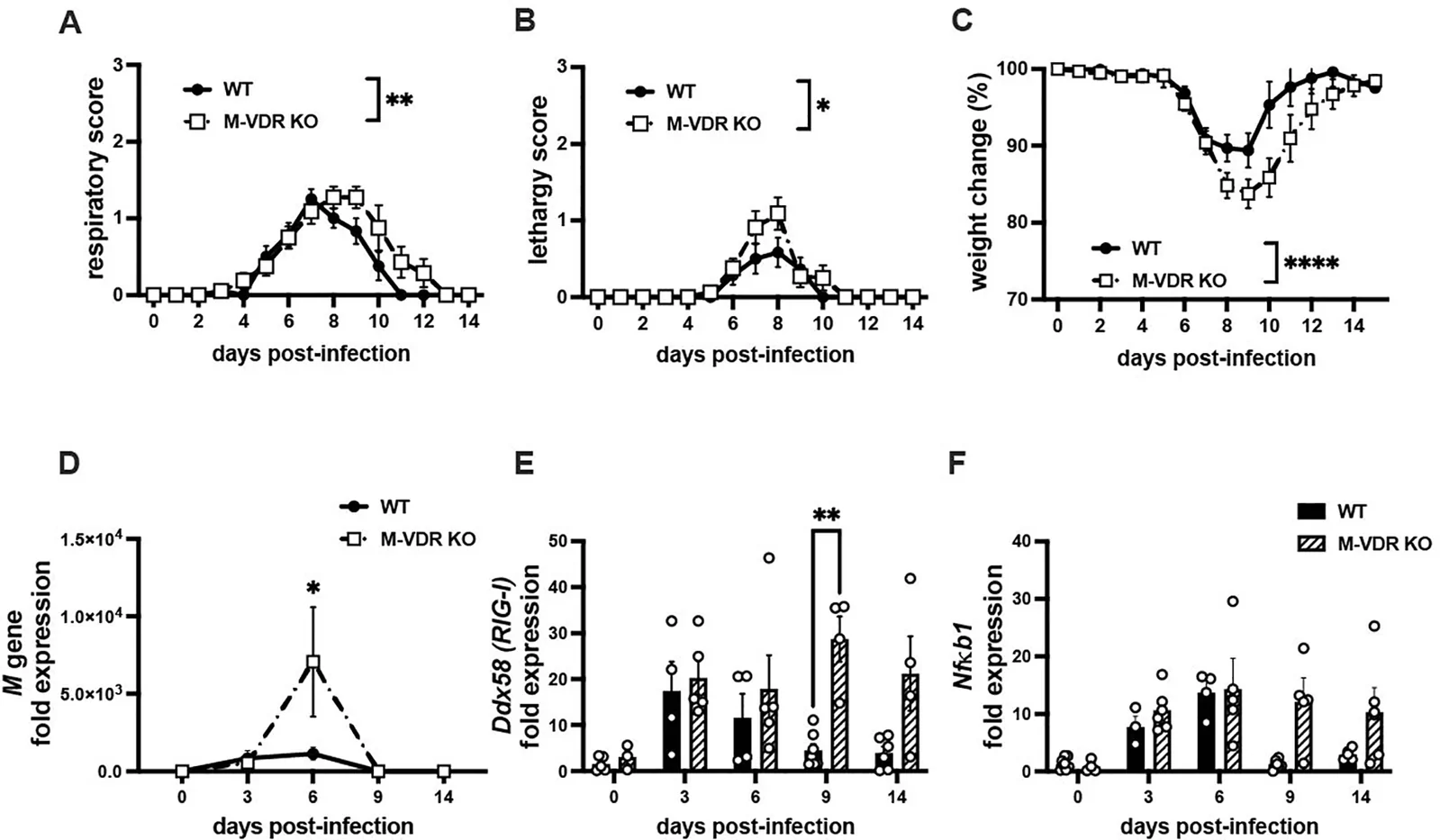
M-VDR KO increases susceptibility to influenza infection. **(A)** Respiratory scores (WT littermate n = 19, M-VDR KO n = 20, 2 experiments), **(B)** lethargy score (WT littermate n = 19, M-VDR KO n = 20, 2 experiments), and **(C)** body weight change (WT littermate n = 19, M-VDR KO n = 20, 2 experiments), **(D)** viral matrix (*M*) gene expression (n = 3 for (d0), n=6 mice/all other time points, 2 experiments) and lung mRNA expression (n = 3–7 mice per group per time point, 2 experiments) of **(E)** *Ddx58 (RIG-I)* and **(F)** *Nfkb1.* Values are the mean ± SEM and represent pooled results from two independent experiments. Each symbol represents an individual mouse. Statistical significance was determined using two-way ANOVA with Tukey’s multiple comparisons test and adjusted using the two-stage step-up Benjamini, Krieger, and Yekutieli FDR procedure. *p < 0.05; **p < 0.01; ****p < 0.0001.

## Discussion

VDR KO mice develop severe influenza virus infection characterized by higher viral loads, increased symptoms, more severe histopathology, and muscle wasting compared to WT controls. The increased severity of influenza in VDR KO mice correlates with the robust immune infiltration and dysregulated cytokine expression in the lungs, particularly at d9 post-infection. The increased mortality of the VDR KO mice was not observed in the M-VDR KO mice at the same influenza A virus dose, suggesting that VDR expression in other cells contributes to the mortality of the full VDR KO mice. PTH treatments of WT mice exacerbated influenza disease outcomes. Demonstrating for the first time that vitamin D status and PTH shape antiviral immunity and pulmonary inflammation following influenza virus infection.

IFN-β is critical for early viral control and the induction of adaptive immunity (28, 29). Severe influenza virus infection of mice corresponded to high type-I interferons and excessive inflammation (30, 31). RIG-I (*Ddx58*) and NF-κB (*Nfkb1*) are central components of the innate immune response to influenza virus, functioning upstream of type I interferon and inflammatory cytokine production. RIG-I serves as a cytosolic sensor of viral RNA and signals through MAVS to activate IRF3/7 and NF-κB, leading to induction of IFN-β and pro-inflammatory mediators (32). VDR KO mice had high *Tnf-α* expression in the lung before infection that remained high throughout the influenza infection. In addition, lungs from VDR KO mice had significantly higher *Ifn-β* at d3 and *Ddx58* at d6 and d9 post-infection than WT littermates. VDR expression is needed to control the amplitude of the early innate viral sensing and downstream inflammatory response to influenza in the lung.

The increased susceptibility of the M-VDR KO mice to influenza, further supports the importance of myeloid cells as vitamin D targets that control the innate and acquired immune response to influenza. Myeloid cell-intrinsic VDR signaling is required for proper regulation of innate antiviral responses and efficient viral clearance. Myeloid cells, including macrophages and inflammatory antigen-presenting cells, are among the first immune cells to recognize influenza virus through pattern recognition receptors such as RIG-I and initiate antiviral cytokine production. Vitamin D has been shown to inhibit NF-κB dependent transcription and limit inflammation (33). Heightened RIG-I and NF-κB associated responses in VDR KO mice contribute to the increased type I interferon response observed early after influenza infection.

Cytokine storms are often the cause of secondary bacterial infections and hospitalization for humans with severe influenza infections (31, 34). The cytokine storms that include IL-17A, TNF-α, IFN-β, IFN-γ etc. play a complex role in viral infections, promoting neutrophil recruitment, anti-viral immunity and cytotoxic T cells but also contributing to lung damage (35, 36). Vitamin D has been shown to decrease IFN-γ and to inhibit the differentiation and proliferation of Th1 cells (37). VDR KO lungs had higher numbers of IFN-γ secreting cells compared to WT lungs following influenza infection. Blocking IFN-γ signaling using anti-IFN-γ therapy significantly alleviated acute lung injury in mice infected with H1N1 influenza virus, demonstrating that excessive IFN-γ contributes to immunopathology (38). VDR KO lungs had higher IL-10 production at d14 post-infection than WT lungs. IL-10 is a key immunoregulatory cytokine that limits inflammation and tissue damage (39). Experimentally, IFN-β treatments have been shown to induce IL-10 secreting Tregs and therefore the increased IL-10 late post-infection could be connected to the high IFN-β at d3 post-infection (40). High *Il-17a*, *Tnf-α, Ifn-β, Ifn-γ* and *Il-10* mRNA expression with more IFN-γ secreting T cells in the VDR KO lungs corresponded with the increased severity of H1N1 influenza symptoms compared to lungs from WT mice. Cytokine dysregulation is a feature of VDR KO mice.

Vitamin D deficiency and VDR KO result in high PTH levels. PTH treatments of WT mice resulted in the increased severity of influenza virus, higher viral M gene and IFN-γ compared to vehicle-treated WT mice. PTH receptors have been demonstrated to be on T cells, and PTH has been shown to induce IL-17A production (19). Patients with chronic kidney disease and hyperparathyroidism have immune dysregulation that results in higher risks of infection (41). CD25 is the alpha chain of the IL-2 receptor and has been shown to be expressed late following activation on conventional T cells and constitutively on regulatory T cells (42). FoxP3 has also been shown to be expressed transiently in activated conventional T cells and stably in Tregs (43). *In vitro* PTH and 1,25(OH)_2_D both induced CD25+ expression on CD4+ T cells and *in vivo* PTH treatments increased FoxP3+ expressing Tregs. Together the results are consistent with the literature that shows that intermittent PTH expands Tregs in humans and mice (44) and the extensive literature that shows that 1,25(OH)_2_D and vitamin D status support both CD4CD25+ and FoxP3+ expressing regulatory T cells (45–50). The effect of VDR KO to increase IL-10 and PTH to induce Tregs, while many inflammatory cytokines are over produced, may seem paradoxical. Perhaps the timing of Treg/IL-10 response is too late to stop the inflammation leading to severe disease in VDR KO mice. IFN-γ is a key antiviral cytokine but also a potent driver of pulmonary inflammation and tissue injury following influenza infection (51, 52). These findings suggest that loss of VDR signaling or elevated PTH shifts the immune response toward exaggerated IFN-γ–mediated inflammation, eliciting a compensatory but delayed regulatory response. It is possible that the daily repeated PTH administration does not adequately model the chronic high PTH levels in VDR KO mice. The daily repeated PTH administrations did not alter serum calcium levels, excluding calcium signaling as a major contributor to influenza severity and demonstrating that intermittent PTH itself contributes to worsened disease outcomes during influenza infection. The finding that intermittent PTH contributes to immune dysfunction, resulting in a severe form of H1N1 influenza virus infection is a novel finding.

There are several limitations to the current study. First, intermittent PTH treatment of WT mice does not fully replicate the chronic PTH elevation observed in VDR KO mice. While osmotic pumps delivering continuous PTH could have been used to mimic chronic elevation, doing so would likely have disrupted mineral homeostasis and induce additional physiological confounders. Nevertheless, the current data still support a distinct effect of intermittent PTH on the host response to influenza. Second, the data supporting the overproduction of cytokines in response to PTH treatment and VDR deletion remain kinetic and correlative. To establish a causal link for the elevated levels of IFN-β1 and IFN-γ, additional experiments utilizing neutralizing antibodies or knockout models are required. However, given the extensive existing literature connecting both the VDR and PTH to increased inflammatory tone, our data strongly support the claims of heightened cytokine production in VDR KO and PTH-treated mice. Third, the influenza M gene was utilized as a surrogate for viral quantification in these experiments. Fortunately, the day 6 data in **Figure 3**—showing that VDR KO mice had both higher M gene expression and a higher load of infectious viral titers (TCID_50_) than WT mice—supports a true difference in viral clearance. Lastly, myeloid cells are highly heterogeneous, and confirming VDR depletion in BMDM does not definitively prove deletion within lung myeloid cells. However, the phenotype of the M-VDR KO mice following influenza infection—characterized by prolonged and elevated M-gene expression alongside more severe influenza symptoms than WT mice—strongly supports a critical role for the VDR in myeloid cells regarding host resistance to influenza.

Together the data support a model in which elevated PTH acts synergistically with the absence of VDR to promote a hyperinflammatory, myeloid and T cell-dominated immune response that fails to clear virus effectively and causes collateral tissue damage. In addition, the data support a critical role for the VDR in myeloid cells that intrinsically controls the innate antiviral response and viral clearance. Vitamin D through the VDR, reduces PTH and restrains the production of multiple cytokines to limit the inflammatory response, with more rapid viral clearance. Vitamin D deficiency is highly prevalent globally, particularly in older adults and individuals with chronic disease that also face higher risk of influenza complications. Vitamin D deficiency may worsen influenza outcomes not only through impaired antiviral defenses, but also because of the effects of PTH that fuels inflammation.

## Data Availability

The raw data supporting the conclusions of this article will be made available by the authors, without undue reservation.
