# Supplementary material for: Myeloid cell expression of the VDR and parathyroid hormone control the host response to influenza infection in mice
Source: Front Immunol. 2026 Sep 30;17:1939052. doi: 10.3389/fimmu.2026.1939052 (PMC13661438; doi:10.3389/fimmu.2026.1939052)
Supplement: Supplementary file 1 [file DataSheet1.docx]

**Supplemental Table I.**

**Primers sequences for Genotyping**

| Mouse Strain | Target allele & Product Size | Forward (5’-3’) | Reverse (5’-3’) |
| --- | --- | --- | --- |
| *VDR Cre* | WT: 229 bp | TGGCTATGCTGAAGGTGCCA | ACTCACCTGAAGAAACCCTT |
|  | Cre: 310 bp | GAACCTGATGGACATGTTCAGG | AGTGCGTTCGAACGCTAGAGCCTGT |
| *VDR Floxed* | WT: 254 bp Floxed: 337 bp | ATGGACAGGAACACACAGCATCA | TCTGACTCCCACAAGTGTACCACGG |
| *LysM Cre* | WT: 350 bp | CTTGGGCTGCCAGAATTTCTC | TTACAGTCGGCCAGGCTGAC |
|  | Cre: 700 bp | CTTGGGCTGCCAGAATTTCTC | CCCAGAAATGCCAGATTACG |

**Primers sequences for qPCR**

| Gene | Forward (5’-3’) | Reverse (5’-3’) |
| --- | --- | --- |
| *M* gene | AGATGAGTCTTCTAACCGAGGTCG | TCGAGATCGGTGTTCTTTCC |
| *Tnf-α* | TGGGAGTAGACA AGGTACAACCC | CATCTTCTCAAAATTCGAGTGACAA |
| *Il-6* | GCTACCAAACTGGATATAATCAGGA | CCAGGTAGCTATGGTACT CCAGAA |
| *Ifn-β* | AGG GCG GAC TTCAAGATC | CTC ATT CCA CCC AGT GCT |
| *Il-17a* | CCTGTCTCTGGCATACACTATTT | CTTCCTGGGACGGAAGTTATATG |
| *Ifn-γ* | TGCATCTTGGCTTGGCAGCTCTTC | GGGTTGACCTCAAACTTGGCA |
| *Il-10* | GGAGAACCTGAAGACCCTCA | GATGTCAAACTCACTCATGGC |
| *Ddx58 (RIG-I)* | GTATCCTCTCCCTCTTCCTCTT | CTGCTTCCCACCTCAATTAGT |
| *Nfkb1* | CGGACTAGAAAGGAGCAAGATG | CGAGGCAAGGACCAGAATAAG |
| *Cyp27b1* | CCGCGGGCTATGCTGGAAC | CTCTGGGCAAAGGCAAACATCTGA |

**Antibodies used for Flow Cytometry**

| Antibodies | Fluorophore | Clone | Manufacturer |
| --- | --- | --- | --- |
| anti-mouse I-A/I-E  (MHCII) | Alexa Fluor 647 | M5/114.15.2 | BD Biosciences |
| anti-mouse CD11b | PE-Cyanine 5 | M1/70 | Invitrogen |
| anti-mouse CD11c | FITC | N418 | Biolegend |
| anti-mouse CD3ε | Alexa Flour 647 | 17A2 | Biolegend |
| anti-mouse NK1.1 | Alexa Flour 700 | S17016D | Biolegend |
| anti-mouse CD19 | Brilliant Violet 421 | 6D5 | Biolegend |
| anti-mouse CD4 | Brilliant Violet 421 | GK1.5 | Biolegend |
| anti-mouse CD8α | PE Cyanine 7 | 53-6.7 | Biolegend |
| anti-mouse IFN-γ | PE Dazzle 594 | XMG1.2 | Biolegend |
| anti-mouse RORγt | PE | B2D | Invitrogen |
| anti-mouse IL-17A | Brilliant Violet 711 | BL168 | Biolegend |
| anti-mouse T-bet | APC | 4B10 | Biolegend |
| anti-mouse FOXP3 | Alexa Flour 488 | MF23 | BD Pharmingen |
| anti-mouse CD25 | PE | PC61 | BD Pharmingen |

**Flow cytometry panels utilized for cell identification.**

| Cell type | Flow cytometry markers |
| --- | --- |
| Myeloid (antigen-presenting) | MHCII^+^CD11b^+^CD11c^+^ |
| NK | CD3^-^CD19^-^NK1.1^+^ |
| CD4 T | CD4^+^CD8^-^ |
| CD8 T | CD4^-^CD8^+^ |
| B cells | CD3^-^CD19^+^NK1.1^-^ |
| Th1^+^/Th17^+^ | CD4+Tbet^+^RORγt^+^, |
| Th1^+^ | CD4^+^Tbet^+^ RORγt^-^ |
| Th17^+^ | CD4^+^Tbet^-^RORγt^+^ |
| Treg | CD4^+^FOXP3^+^ |

**Supplemental Figure 1. Sex-based analysis of influenza disease outcomes in WT and VDR KO mice.** Survival of A) WT mice and B) VDR KO mice, body weight change in C) WT mice and D) VDR KO mice, respiratory scores E) WT mice and F) VDR KO mice, lethargy scores in G) WT mice and H) VDR KO mice. Values are the mean ± SEM. WT female n = 16, WT male n = 20, VDR KO female n = 13, and VDR KO male n = 15, as indicated in each panel. Statistical significance was assessed using two-way ANOVA for (C-H), and log-rank test for survival (A,B). ns, not significant; *** p < 0.001.

**Supplemental Figure 2. Serum calcium levels, muscle weight loss and influenza-specific antibody responses following infection.** A) Serum calcium concentrations in WT, VDR KO, and WT mice treated with PTH (n=5-9), B) gastrocnemius muscle mass (gastroc.wt) per g of body weight (bw) from VDR KO mice at d0 and d9 post-influenza infection (n=3-5), C) influenza-specific serum IgG levels measured by ELISA (n=6/group). Each symbol represents an individual mouse. Values are the mean ± SEM. Statistical significance was assessed using an unpaired Student’s t-test. No statistically significant differences were observed between the groups.

**Supplementary Figure 3. Representative flow cytometry gating strategy for CD4⁺IFN**-γ⁺ **T cells.** Single-cell suspensions from lung tissue were initially gated on lymphocytes based on forward scatter (FSC) and side scatter (SSC), followed by exclusion of doublets. CD3⁺ T cells were then gated and further separated into CD4⁺ and CD8⁺ T-cell subsets. IFN-γ-producing CD4⁺ T cells were quantified within the CD3⁺CD4⁺ population as CD3⁺CD4⁺IFN-γ⁺ cells.

**Supplemental Figure 4. Influenza virus symptoms and immune cell infiltration in PTH-treated and vehicle-treated WT mice.** WT mice were treated with vehicle or PTH and infected with H1N1 influenza virus. A) Survival, B) percent body weight loss, and frequencies of C) myeloid APC, D) NK, E) B, F) CD4⁺ T, and G) CD8⁺ T cells in the lungs. Values are the mean ± SEM from two independent experiments (n = 3–6 mice per timepoint in each of the two groups). Each symbol represents an individual mouse. Survival was analyzed using the log-rank (Mantel–Cox) test, and immune cell frequencies and weight change were analyzed using two-way ANOVA with Tukey’s multiple comparisons test. ** p < 0.01; **** p < 0.0001.

**Supplemental Figure 5. Effects of PTH on immune cells following influenza virus infection in vivo or in vitro from anti-CD3 stimulation of splenocytes.** A) IFN-γ producing cell counts, frequency of B) CD4⁺IFNγ⁺ T cells, C) CD8⁺IFNγ^+^ T cells, D) Th1⁺ cells, E) Th1⁺Th17^+^ cells, F) Th17⁺ cells G) IL-17A+ cells, and H) Tregs in lungs of WT and PTH-treated WT mice (n = 3-6 group) Each symbol represents an individual mouse. I) CD4⁺CD25⁺ T cell frequencies following activation in vitro with anti-CD3 and PTH or 1,25(OH)_2_D for 48h. Values represent the mean ± SEM, pooled from one independent experiment, n = 3-6 mice/group. Two-way ANOVA with Tukey’s multiple comparison test was used to assess statistical significance. * p < 0.05; ** p,0.01; *** p < 0.001; **** p < 0.0001**.**

**Supplemental Figure 6. WT, M-VDR KO and VDR KO mice.** A) Immunoblot for VDR protein expression in the kidney and macrophage from the bone marrow (BMDM) of WT, M-VDR KO and VDR KO mice. The BMDM have a nonspecific higher MW band present in all samples. The specific VDR band (53 kDa) is indicated by the red arrow. GAPDH (34 kDa) was used as a loading control. WT mice have VDR protein expression in kidney and BMDM, while the M-VDR KO mice had VDR protein in the kidney but not the BMDM and VDR KO mice had no VDR protein in either the kidney or BMDM. B) Survival of WT and M-VDR KO mice following influenza infection. Lung cytokine mRNA expression for C) *Ifn-β*, D) *Tnf-α*, E) *Ifn-γ*, and F) *Il-6* was measured by qPCR. Fold change is shown relative to uninfected controls (d0). Values are the mean T± SEM (n = 3-6 mice per treatment and in each group). Each symbol represents an individual mouse. Survival was analyzed using the log-rank (Mantel-Cox) test, and cytokine expression was analyzed using two-way ANOVA with Tukey’s multiple comparisons test.
